# Magnetic resonance imaging in polymyalgia rheumatica—contrast enhancement is not always needed

**DOI:** 10.1007/s00393-023-01394-7

**Published:** 2023-08-11

**Authors:** Martin Fruth, Annika Seggewiss, Jessica Kozik, Philipp Martin-Seidel, Xenofon Baraliakos, Jürgen Braun

**Affiliations:** 1 Evidia Radiologie am Rheumazentrum Ruhrgebiet, Claudiusstr. 45, 44649 Herne, Germany; 2 Rheumazentrum Ruhrgebiet, Herne, Germany

**Keywords:** Magnetic resonance imaging, Inflammation, Polymyalgia rheumatica, Pelvic girdle, Diagnosis, Magnetresonanzbildgebung, Entzündung, Polymyalgia rheumatica, Beckengürtel, Diagnose

## Abstract

**Background:**

Extracapsular inflammation at entheseal sites in the pelvic girdle as demonstrated by magnetic resonance imaging (MRI) was shown to be useful as an additional tool for diagnosing polymyalgia rheumatica (PMR). However, it is unclear whether MRI needs to be performed with contrast enhancement or whether oedema-sensitive sequences are sufficient.

**Objective:**

To evaluate the performance of T2w TIRM (turbo inversion recovery magnitude) imaging compared to fat-saturated contrast-enhanced (ce) T1w at predefined pelvic sites to detect extracapsular inflammation in patients with PMR.

**Methods:**

A total of 120 pelvic MRIs of patients with pelvic girdle pain, 40 with clinically diagnosed PMR and 80 controls, were retrospectively scored by three blinded radiologists separately evaluating the MRI with and without contrast enhancement at 19 previously defined pelvic structures. The intra- and interrater reliability and the diagnostic performance of both techniques were statistically analysed and evaluated.

**Results:**

The detection of inflammatory MRI signals correlated moderately between both techniques (Cohen’s κ 0.583). With ceT1w imaging 20.7% more sites were detected as inflamed compared to T2w TIRM in PMR patients. Inter- and intrareader reliability was superior with ceT1w imaging. If the inflammatory signal was detected at three sites bilaterally including the origin of the rectus femoris muscle or adductor longus muscle, the sensitivity and specificity was 100% and 97.1% by ceT1w imaging vs. 80.8% and 93.3% by T2w TIRM, respectively.

**Conclusion:**

Contrast enhancement is superior to oedema-sensitive MRI in the detection of extracapsular inflammation in PMR. However, using T2w TIRM also detects many but not all PMR cases.

## Abstract (de)

**Hintergrund:**

Der Nachweis einer extrakapsulären Entzündung an Muskel- oder Sehnenansatzstellen im Beckenbereich mittels Magnetresonanztomographie (MRT) hat sich als hilfreich im Sinne eines zusätzlichen Instruments zur Diagnose einer Polymyalgia rheumatica (PMR) herausgestellt. Jedoch ist nicht klar, ob die MRT mit Kontrastverstärkung durchgeführt werden muss oder ob ödemsensitive Sequenzen ausreichend sind.

**Ziel:**

Ziel der vorliegenden Studie war es, die Aussagekraft der T2w-TIRM(Turbo-Inversion-Recovery-Magnitude)-Bildgebung im Vergleich zur fettgesättigten kontrastverstärkten T1w-Bildgebung an prädefinierten Stellen des Beckens zu untersuchen, um extrakapsuläre Entzündungsbereiche bei Patienten mit PMR zu diagnostizieren.

**Methoden:**

Retrospektiv klassifiziert wurden 120 Becken-MRT-Aufnahmen von Patienten mit Schmerzen im Beckenbereich, 40 mit klinisch diagnostizierter PMR und 80 Kontrollen; zu diesem Zweck beurteilten 3 Radiologen verblindet separat die MRT-Aufnahmen mit und ohne Kontrastverstärkung an 19 vorher festgelegten Beckenstrukturen. Die Intra- und Inter-Rater-Reliabilität sowie die diagnostische Leistung beider Techniken wurden statistisch analysiert und beurteilt.

**Ergebnisse:**

Die Erkennung inflammatorischer MRT-Signale war im Vergleich zwischen den beiden Techniken moderat korreliert (Cohen‑κ 0,583). Mittels kontrastverstärkter T1w-Bildgebung wurden bei PMR-Patienten 20,7% mehr Stellen im Sinne einer entzündlichen Veränderung erkannt als mit der T2w-TIRM-Bildgebung. Die Inter- und Intra-Rater-Reliabilität war mit der kontrastverstärkten T1w-Bildgebung höher. Wenn das inflammatorische Signal bilateral an 3 Stellen einschließlich des Ursprungs des M. rectus femoris oder des M. adductor longus erkannt wurde, betrug die Sensitivität 100% und die Spezifität 97,1% bei der kontrastverstärkten T1w-Bildgebung vs. 80,8% bzw. 93,3% bei der T2w-TIRM-Bildgebung.

**Schlussfolgerung:**

Die Kontrastverstärkung ist der ödemsensitiven MRI bei der Erkennung extrakapsulärer Inflammation bei PMR überlegen. Allerdings werden auch mit der T2w-TIRM-Bildgebung viele, jedoch nicht alle Fälle mit PMR erkannt.

## Introduction

Polymyalgia rheumatica (PMR) is a prevalent rheumatic disease in patients older than 50 years of age and is associated with pain and stiffness, most often affecting the pelvis, hips, legs, neck, shoulders and upper arms [1]. The proposed classification criteria [2, 3] have mainly relied on clinical findings but European Alliance of Associations for Rheumatology (EULAR) imaging [4] and treatment recommendations [5] were recently published.

Although visualisation of extracapsular inflammation by magnetic resonance imaging (MRI, [6–12]) and ^18^F‑fluorodeoxyglucose positron emission tomography with computed tomography (FDG-PET/CT) have been shown to provide characteristic imaging findings suggestive of additional diagnostic value for PMR [13–21], imaging is currently not recommended in patients with a suspected diagnosis of PMR [22].

Nevertheless, inflammatory changes described to be typical for PMR are, as recently shown [11, 12], displayed as contrast enhancement around tendinous and capsular structures that most likely reflect inflammation of the peritendineum and the outer neurovascular layer of fibrous capsules rather than the synovium making synovitis a mere secondary phenomenon [6, 11, 12]. However, these MRI findings have not been histopathologically confirmed to date.

The inflammatory pattern in the pelvic girdle described to be characteristic for PMR usually consists of numerous bilateral symmetric peritendinitis and pericapsulitis with rather constant involvement of the tendinous origins of the rectus femoris or adductor longus muscles. In a previous study, using contrast-enhanced (ce)MRI bilateral inflammation of four tendinous and capsular sites including proximal rectus femoris or adductor longus muscle origin were very characteristic for PMR and discriminated convincingly between PMR cases and controls [12]. Similar observations were recently published for the shoulder girdle [23].

In clinical routine, however, musculoskeletal MRI beyond specialised rheumatologic imaging is usually and often targeted to structural, i.e. degenerative changes, and is therefore often performed without contrast enhancement. In patients with suspected PMR, especially in the nonspecialised outpatient setting, contrast-free MRI scans of the shoulder or pelvic girdle are often performed to rule out other reasons explaining joint and muscle pain [24]. Using fat-saturated T2-weighted (T2w) MRI extracapsular inflammation is displayed as peritendinous and pericapsular oedema [6, 12].

**Fig. 1:**
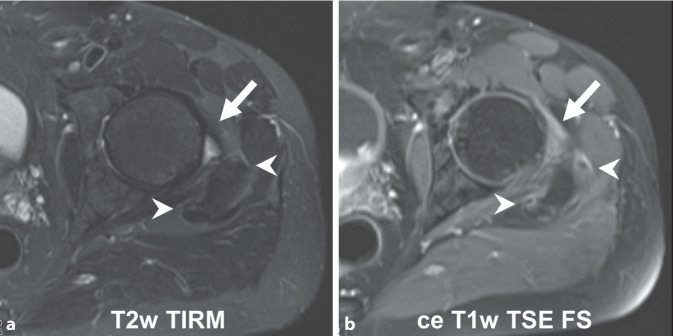
Oedema-sensitive T2-weighted turbo inversion recovery magnitude (TIRM, **a**) and contrast-enhanced fat-saturated T1-weighted turbo echo spin (TSE FS, **b**) on the level of hip joint. Pericapsular inflammation (*arrow*) as shown by contrast-enhanced magnetic resonance imaging (ceMRI) lacks a correlate by oedema-sensitive imaging. Peritendinous inflammation around glutaeus medius and minimus tendons (*arrow heads*) correlate with only minute oedema, which can easily be overlooked

Based on our experience with PMR and MRI we observed that clearly detectable peritendinous contrast enhancement frequently correlates with only limited or even no peritendinous oedema (Fig. 1). Therefore, noncontrast-enhanced MRI scans are likely to underestimate the extent of inflammatory lesions with the consequence of misinterpretation of this finding.

In this retrospective study we examined peritendinous and pericapsular inflammation in the pelvic girdle by comparing two MRI techniques, one with contrast enhancement and one concentrating on fat saturation without using a contrast agent. The aim was to assess whether the use of contrast agent is necessary to provide evidence for the presence of MRI typical changes at predefined sites in the pelvis of patients with PMR compared to controls.

## Patients and methods

### Patients

For the purpose of this study all MRI scans of 120 patients with pelvic girdle pain (40 clinically diagnosed with PMR) described in our previous study [12] were revaluated with regard to the comparison between the two MRI techniques T1-weighted turbo spin echo (T1w TSE) before and after application of gadolinium (contrast-enhanced fat-saturated T1w turbo spin echo, ceT1w TSE FS), and the oedema-sensitive T2-weighted turbo inversion recovery magnitude (T2w TIRM) sequence [11].

A third of these patients were diagnosed with new onset PMR (*n* = 40), of whom 10 were reclassified as PMR-like onset rheumatoid arthritis (RA) later since they developed peripheral arthritis during the further course of disease. The remaining 80 patients had other inflammatory or non-inflammatory causes to explain their pain. All cases were diagnosed by an expert rheumatologist including all PMR and PMR-like onset RA cases. All PMR cases fulfilled the 1979 criteria for PMR [2] and most patients also fulfilled the 2012 preliminary criteria for PMR [3]. Patient demographics and characteristics have been published previously [12] but are available in Table 1.

**Table 1:** Characteristics of polymyalgia rheumatica (PMR) cases and controls

	PMR	Controls
Quantity, *n*	40	80
Age (years), mean (SD)	64.2 (9.0)	64.1 (8.8)
Male, *n* (%)	18 (45)	30 (37.5)
C‑reactive protein (mg/dl), mean (SD)	3.48 (4.02)	0.44 (0.47)
ESR (mm/h), mean (SD)	36.6 (21.9)	13.8 (9.4)
Rheumatoid factor positive (≥ 14 IU/ml), *n *(%)	3 (7.5%)	16 (20.0%)
CCP antibody positive (≥ 40 IU/ml), *n* (%)	2 (5.0%)	10 (12.5%)
Main diagnosis, *n* (%)	PMR 30 (75.0%)	Degenerative disc or joint disease 21 (26.3%)
	PMR like onset RA 10 (25%)	Rheumatoid arthritis 21 (26.3%)
		Axial spondyloarthritis 16 (20%)
		Fibromyalgia 7 (8.8%)
		Connective tissue diseases such as SLE 5 (6.3%)
		Psoriatic arthritis 4 (5%)
		Insufficiency fracture 2 (2.5%)
		SAPHO-Syndrome 1 (1.3%)
		DISH-Syndrome 1 (1.3%)
		CPPD arthritis 1 (1.3%)
		Osteomalacia 1 (1.3%)

*SD* standard deviation, *ESR* erythrocyte sedimentation rate, *CCP* cyclic citrullinated peptide, *PMR* polymyalgia rheumatica, *RA* rheumatoid arthritis, *SLE* systemic lupus erythematosus, *SAPHO* synovitis acne pustulosis hyperostosis osteitis, *DISH* diffuse idiopathic skeletal hyperostosis, *CPPD* calcium pyrophosphate dihydrate

### MRI

All pelvic MRI scans consisted of T1-weighted turbo spin echo (T1w TSE), oedema-sensitive T2-weighted turbo inversion recovery magnitude (T2w TIRM) and contrast-enhanced fat-saturated T1-weighted turbo spin echo (ceT1w TSE FS) after intravenous application of gadolinium-based contrast agent gadoteric acid in coronal and transversal planes with exact alignment and thickness of the slices. The scans typically covered the pelvis from the level of segment L4/5 to the subtrochanteric proximal femur in the transverse plane, whereby the coronal plane typically exceeded this a little. A total of 106 patients were examined with Siemens (Siemens Healthineers, Erlangen, Germany) Aera, 10 with Siemens Avanto and 4 with Siemens Skyra. Scanning parameters differed slightly due to individual adjustments and different field strengths. Detailed parameters previously published [11] are available in Table 2.

**Table 2:** Overview of the sequence parameters

	TR (ms)	TE (ms)	TI (ms)	Fat saturation	FOV (mm)	Matrix	Slice thickness (mm)/gap	Gd contrast
*Siemens Aera 1.5* *T*								
T1w TSE coronal	511	10	–	–	400 × 400	320 × 320	6/20%	–
T1w TSE transversal	526	13	–	–	400 × 250	240 × 384	6/20%	–
T2w TIRM coronal	3550	56	160	Inversion	400 × 400	320 × 320	6/20%	–
T2w TIRM transversal	4400	59	160	Inversion	400 × 250	384 × 240	6/20%	–
T1w TSE FS coronal	477	8.3	–	Spectral	400 × 400	320 × 320	6/20%	Yes
T1w TSE FS transversal	476	11	–	Spectral	400 × 250	200 × 320	6/20%	Yes
*Siemens Skyra 3.0* *T*								
T1w TSE coronal	550	10	–	–	380 × 380	448 × 448	4/20%	–
T1w TSE transversal	550	11	–	–	350 × 262.5	448 × 336	5/20%	–
T2w TIRM coronal	5860	55	220	Inversion	380 × 380	448 × 448	4/20%	–
T2w TIRM transversal	5040	57	220	Inversion	380 × 285	384 × 288	5/20%	–
T1w TSE FS coronal	635	10	–	Spectral	380 × 380	448 × 448	4/20%	Yes
T1w TSE FS transversal	680	11	–	Spectral	350 × 262.5	448 × 336	5/20%	Yes
*Siemens Avanto 1.5* *T*								
T1w TSE coronal	490	12	–	–	400 × 400	768 × 768	5/30%	–
T1w TSE transversal	583	10	–	–	370 × 307	786 × 636	6/20%	–
T2w TIRM coronal	3500	57	140	Inversion	400 × 400	320 × 320	5/30%	–
T2w TIRM transversal	5100	63	160	Inversion	370 × 296	320 × 256	6/20%	–
T1w TSE FS coronal	565	12	–	Spectral	400 × 400	640 × 640	5/30%	Yes
T1w TSE FS transversal	590	11	–	Spectral	370 × 300	256 × 208	6/20%	Yes

*TSE* turbo spin echo, *TIRM* turbo inversion recovery magnitude, *FS* fat saturation, *TR* repetition time, *TE* echo time, *TI* inversion time, *FOV* field of view, *Gd* Gadolinium

### Investigated sites and image analysis

For the revaluation, the contrast-enhanced sequences of each examination were erased from the examinations resulting in two independent MRI sets—the new virtually contrast-free set and the old set with the remaining contrast-enhanced images. Both sets of MRI scans underwent a new pseudonymization to provide blinding of the raters regarding demographic, clinical and biometric information. In an analogous manner to the previous study on contrast-enhanced pelvic MRI the images were scored by the same three radiologists regarding the evaluation of contrast enhancement and now also of oedema around the 19 predefined tendinous and capsular structures in coronal and transversal images. A total of 10 PMR cases and 20 controls were read twice in both sets to evaluate the intrarater reliability, resulting in 150 contrast-enhanced MRIs and 150 oedema-sensitive MRI scans for each reader. The raters had no access to the contrast-enhanced images while reading oedema-sensitive images.

Since the T2w TIRM and ceT1w FS images in all MRI scans were aligned exactly to the same position an anatomically proper comparison of all investigated sites in all patients was possible.

The following predefined anatomic sites of interest were used:

- Unilateral:
  - the lumbar interspinous bursae and paraspinous origins of deep spinal musculature
- Bilateral:
  - around the superior anterior iliac spine and anterior iliac crest representing various muscle origins like the abdominal wall musculature, including tensor fasciae latae and sartorius muscles,
  - around the proximal origin of the straight and reflected head of the tendon of the rectus femoris muscle at the anterior inferior iliac spine and supraacetabular ridge,
  - around the distal part of the gluteus medius and minimus muscle tendon at the trochanteric insertion,
  - around the fibrous hip capsule at the level of the femoral neck,
  - around the tendon of the obturator internus muscle at its reflection at the posterior margin of the ischium,
  - around the adductor longus muscle tendon origin at the inferomedial pubic symphysis,
  - around the distal iliopsoas muscle tendon at the lower trochanter,
  - around the common ischiocrural origin (hamstring) at the ischial tuberosity,
  - around the distal insertional site of the gluteus maximus muscle at the gluteal tuberosity.

All sites were scored binary, absence or presence of oedema- or contrast-enhancement regardless of individual amount. To be scored positive a circumferential oedema or contrast enhancement at these sites had to be visible in two contiguous slices in one plane or on two perpendicular planes. The raters only scored absence or presence of oedema or contrast enhancement at individual sites but made no diagnostic decision regarding the different tests mentioned below.

### Statistical analyses

Inter- and intrarater reliability were calculated as κ correlations for each reading point in the oedema-sensitive and contrast-enhanced MRI set.

The congruity between detection of inflammation by peritendinous oedema versus contrast enhancement was analysed for each rater at every evaluated site and displayed as relative divergence and as κ correlations from the contrast-enhanced study as reference standard. Sites with highest incongruity were identified.

Finally, the detection of inflammation at a varying number of bilaterally involved sites including origins of the rectus femoris or adductor longus muscles has been evaluated as a confirming imaging test by contrast-enhanced and oedema-sensitive imaging. For the tests the individual results of all three raters were pooled. Receiver operating characteristic curves and optimal test criteria were calculated.

Descriptive demographic and clinical data are presented as mean ± standard deviation (SD) when referring to quantitative variables and as absolute frequencies and percentages when referring to qualitative variables. The McNemars test was used to compare the proportion of discrepancy between decision on oedema-sensitive and contrast-enhanced imaging at individual sites, for this purpose decisions for both sides of a site were grouped together, i.e. right and left proximal rectus femoris origins. The Mann–Whitney U test was used to compare the data between subgroups. A value of *p* < 0.05 was considered statistically significant. Statistical analyses were performed using SPSS version 23 (IBM, Armonk, NY USA).

## Results

### Inter- and intrarater reliability

The average pairwise agreement between the three raters for all 2280 evaluated sites was 88.3% (Fleiss κ 0.748) in contrast-enhanced imaging and 84.0% (Fleiss κ 0.614) in oedema-sensitive imaging, while pairwise agreements ranged from 86.7 to 90.7% (Cohen’s κ 0.713–0.805) with contrast-enhanced MRI and 81.1 to 86.4% (Cohen’s κ 0.565–0.655) in the oedema-sensitive set.

Rating of contrast-enhanced images resulted in almost perfect intrareader agreements of 91.4–94.2% (Cohen’s κ 0.818–0.881), while it decreased to 88.2–93.1% (Cohen’s κ 0.751–0.836) in oedema-sensitive imaging.

### Correlation between oedema-sensitive and contrast-enhanced imaging

Regarding the detection of inflammation by oedema-sensitive versus contrast-enhanced MRI sequences an incongruent decision was detected in 18.6% of sites. In detail, 13.2% of sites were identified as inflamed by contrast-enhanced imaging and 5.4% sites were rated as inflamed by oedema-sensitive sequences but showed no contrast enhancement. One-to-one correlation of every evaluated site by all raters showed only moderate agreement between oedema-sensitive and contrast-enhanced imaging with Cohen’s κ of 0.583 (range 0.546–0.611 for the individual raters).

In the PMR group 20.7% more sites were rated as inflamed by contrast-enhanced imaging compared to a yield of only 9.5% more in the control group.

Except for the hamstring tendon origins and the tendinous insertion of the gluteus medius and minimus muscles with 4.2% each, considerably more inflammation at all other investigated sites was detected by contrast-enhanced imaging in the PMR group ranging from 18.3% at the anterior superior iliac spine up to 34.6% around the hip capsule. In about 73% of all MRI scans of the PMR group, more extracapsular inflammation was detected by contrast-enhanced imaging.

**Fig. 2:**
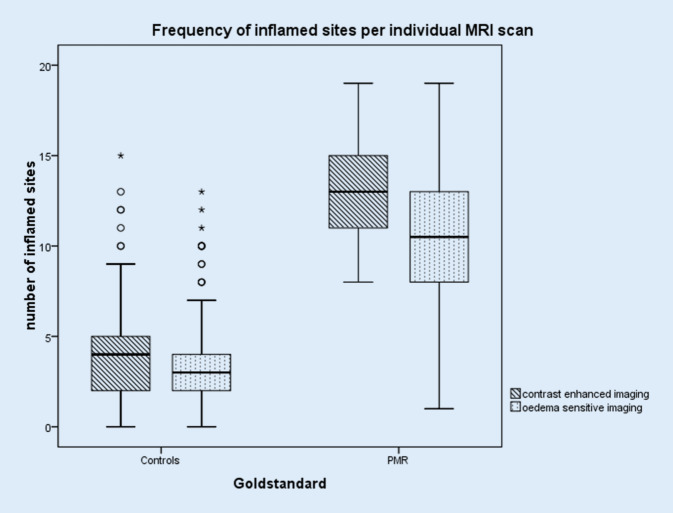
Frequency of inflamed sites per individual magnetic resonance imaging (MRI) scan in controls and polymyalgia rheumatica (PMR) cases as observed by contrast-enhanced versus oedema-sensitive imaging

Furthermore, ceMRI signals of inflammation were seen in 13.4 ± 2.8 sites in the PMR vs. 3.9 ± 2.4 in the control group, while less inflammation was found in the oedema-sensitive MRI: 10.4 ± 3.7 sites in the PMR group vs. 3.1 ± 2.4 in the control group. Moreover, the range of individual counts of inflamed sites per MRI scan in the PMR group was greater in oedema-sensitive imaging: 1–19 inflamed sites, compared to contrast-enhanced imaging with 8–19 inflamed sites (Fig. 2).

### Comparisons between the two MRI techniques

**Fig. 3:**
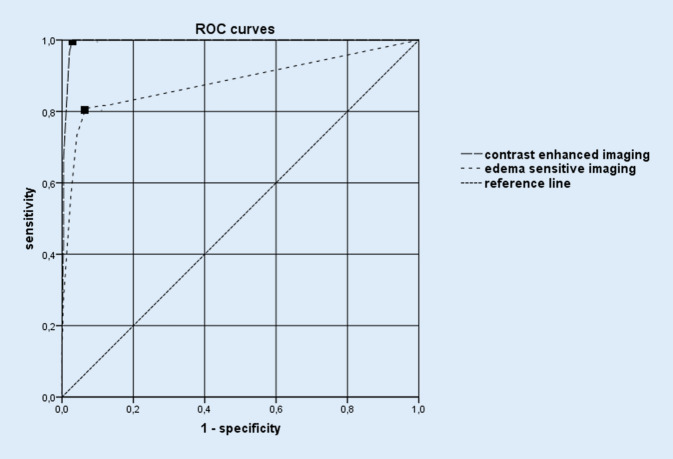
Receiver operator characteristic (ROC) curves of oedema-sensitive and contrast-enhanced imaging. A varying number of bilaterally inflamed sites including at least a rectus femoris or adductor longus muscle origin is hypothesized as a confirmatory imaging test

Performance of T2w TIRM imaging was inferior to fat-saturated contrast-enhanced T1w if a varying number of bilaterally inflamed sites including rectus femoris muscle and adductor longus muscle origins was taken as gold standard for the diagnosis of PMR. If peritendinous or pericapsular inflammation as shown by contrast enhancement was present at three sites bilaterally of which one site was the origin of the proximal rectus femoris or adductor longus muscle, PMR cases were differentiated best from controls with a sensitivity of 100% and specificity of 97.1%, area under the curve (AUC) 0.994 (Fig. 3). The same criteria also provided the best performance in oedema-sensitive imaging, but the sensitivity and specificity were lower than what was achieved for ceMRI: 80.8–93.3%, AUC 0.882. On average 7.7 more patients with PMR were identified by ceMRI compared to oedema-sensitive MRI. Thus, every 5th patient suspected of having PMR would have to undergo a second MRI using a contrast agent if the oedema-sensitive MRI was negative.

## Discussion

In this study we show that the ability of fat-saturated contrast-enhanced T1w imaging is in general superior to oedema-sensitive T2w TIRM imaging in the detection of extracapsular inflammation as a potentially discriminating imaging marker for PMR. Furthermore, we analysed the congruity of inflammation detected by these different MRI techniques. To our knowledge this is the first study that compares oedema-sensitive and contrast-enhanced MRI in PMR.

In our previous study we described and defined a stereotypic pattern of extracapsular inflammation in contrast-enhanced pelvic girdle MRI of patients with PMR and concluded that bilateral inflammation of four extracapsular sites in the pelvic girdle including either the rectus femoris or adductor longus muscle origin could be used as a diagnostic test to identify PMR [12]. Here in this revaluation with all MRI rated a second time by radiologists blinded to clinical data we confirm the high diagnostic capability of contrast-enhanced MRI in PMR by showing that cases were discriminated from controls with a sensitivity of 100% and a specificity of 97.1% if inflammation was visible at only three bilateral sites in the pelvic girdle including one key site. All tests performed differed only slightly from our previous results clearly confirming that the characteristic imaging feature of PMR in pelvic girdle MRI is a bilateral inflammation at the key site, rectus femoris or adductor longus muscle origin—in combination with other bilaterally inflamed extracapsular sites. Bilateral inflammation of a key site alone would already discriminate with a sensitivity and specificity of 100% and 95.0%, respectively, while the addition of a varying number of more bilaterally involved sites would mainly contribute to specificity.

Oedema-sensitive imaging correlated moderately with contrast-enhance imaging. Thus, there were 20.7% fewer inflammatory changes detected in the PMR group. Moreover, the reproducibility of oedema-sensitive imaging was inferior to contrast-enhanced imaging as expressed by inferior intra- and interrater correlations.

However, if oedema-sensitive MRI revealed bilateral inflammation at three sites including a key site, patients with PMR were identified with a sensitivity of 80.8% and specificity of 93.3%. In other words, in roughly only 1 out of 5 patients contrast-enhanced MRI will be of additional value. Taking into account the downsides of intravenous gadolinium-based contrast agent use, such as the additional time and effort needed for the longer scanning protocol and the patients’ discomfort by the inevitable intravenous cannula, routine application of a contrast agent does not seem necessary in most pelvic MRI scans when searching for a PMR pattern of inflammation. However, if the presumptive diagnosis of PMR is not confirmed by oedema-sensitive imaging additional application of a contrast agent is likely to increase the diagnostic gain.

No study has so far directly compared the performance of MRI and 18F-FDG PET-CT to provide additional evidence for a diagnosis of PMR. The high sensitivity and specificity values reported for ceMRI suggest that it is as least as good as the technique using radiation with an effective dose after i.v. application of 350 MBq FDG leading, according to the Medical Internal Radiation Dose (MIRD) Committee, to at least 6.7 mSv, possibly reaching 14–18 mSV [24]. This exposure, however, can be minimized by using, for example, low-dose CT. There is a theoretical advantage of using 18F-FDG PET-CT for a potential discovery of malignoma which may mimic PMR symptoms [21]. However, malignomas are rather rarely discovered when searching for the cause of PMR symptoms. Nevertheless, even one case detected early may be reason enough to perform 18F-FDG PET-CT but it is unclear whether additional clinical symptoms cannot provide guidance as to which imaging technique to perform. The value of 18F-FDG-PET/CT in identifying the cause of fever of unknown origin (FUO) and inflammation of unknown origin has been recently established [25].

Finally, we like to stress that there is evidence that the characteristic inflammatory MRI signals can also help to discriminate patients with PMR at other sites including rheumatoid arthritis [26] and that these signals will likely vanish after successful therapy [27, 28].

## Conclusion

Contrast-enhanced pelvic magnetic resonance imaging (MRI) uncovers more extracapsular inflammatory lesions in polymyalgia rheumatica (PMR). It is more reliable than oedema-sensitive MRI, but oedema-sensitive MRI shows the typical pattern similarly in most cases. Therefore, application of contrast agents is only necessary if oedema-sensitive imaging is not sufficient to show the typical MRI pattern.

### Key messages.

- Both contrast-enhanced and oedema- sensitive MRI sequences such as STIR show characteristic findings of pelvic structures in PMR.
- Assessment of extracapsular inflammation by MRI in PMR is more reliable and sensitive when contrast enhancement is used.
- If oedema-sensitive MRI is not sufficient to confirm PMR contrast agents should be considered.

## Declarations

This retrospective study was performed after consultation with the institutional ethics committee and in accordance with national legal requirements.
